# Free Energy Barriers for Passive Drug Transport through the *Mycobacterium tuberculosis* Outer Membrane: A Molecular Dynamics Study

**DOI:** 10.3390/ijms25021006

**Published:** 2024-01-13

**Authors:** Ilya S. Steshin, Alexander V. Vasyankin, Ekaterina A. Shirokova, Alexey V. Rozhkov, Grigory D. Livshits, Sergey V. Panteleev, Eugene V. Radchenko, Stanislav K. Ignatov, Vladimir A. Palyulin

**Affiliations:** 1 Department of Chemistry, Lobachevsky State University of Nizhny Novgorod, Nizhny Novgorod 603022, Russia; ilya.steshin@icloud.com (I.S.S.); alexandrvasyankin@gmail.com (A.V.V.); ekashirokova@gmail.com (E.A.S.); rozhkov@chem.unn.ru (A.V.R.); grigory.livshits@gmail.com (G.D.L.); pasv1984@gmail.com (S.V.P.); genie@qsar.chem.msu.ru (E.V.R.); 2 Department of Chemistry, Lomonosov Moscow State University, Leninskie Gory 1/3, Moscow 119991, Russia

**Keywords:** tuberculosis, mycobacterium, cell wall, outer membrane, drug permeability, passive transport, molecular dynamics, activation barrier, free energy profile, potential of mean force

## Abstract

The emergence of multi-drug-resistant tuberculosis strains poses a significant challenge to modern medicine. The development of new antituberculosis drugs is hindered by the low permeability of many active compounds through the extremely strong bacterial cell wall of mycobacteria. In order to estimate the ability of potential antimycobacterial agents to diffuse through the outer mycolate membrane, the free energy profiles, the corresponding activation barriers, and possible permeability modes of passive transport for a series of known antibiotics, modern antituberculosis drugs, and prospective active drug-like molecules were determined using molecular dynamics simulations with the all-atom force field and potential of mean-force calculations. The membranes of different chemical and conformational compositions, density, thickness, and ionization states were examined. The typical activation barriers for the low-mass molecules penetrating through the most realistic membrane model were 6–13 kcal/mol for isoniazid, pyrazinamide, and etambutol, and 19 and 25 kcal/mol for bedaquilin and rifampicin. The barriers for the ionized molecules are usually in the range of 37–63 kcal/mol. The linear regression models were derived from the obtained data, allowing one to estimate the permeability barriers from simple physicochemical parameters of the diffusing molecules, notably lipophilicity and molecular polarizability.

## 1. Introduction

Tuberculosis (TB) is a severe disease caused by a single bacterial agent, *Mycobacterium tuberculosis* (Mtb). Currently, TB is one of the most widespread and socially significant infections that constitutes the leading cause of mortality from a single pathogen (exceeding the mortality of AIDS and malaria) [1,2]. In 2021, 1.6 million people died from TB worldwide [1], and it is estimated that the number of individuals with the latent form of TB infection is up to 2 billion people around the world [3,4]. Particularly dangerous is the emergence of Mtb strains with multi-drug resistance (MDR) and extensive drug resistance (XDR) against known antibiotics. This is a new challenge for modern medicine, which urgently requires the creation of new antibacterial agents. Massive efforts are being made worldwide to develop new antituberculosis drugs using both experimental and theoretical methods. However, the development of such agents is hindered by the fact that many active compounds cannot permeate the extremely complex and strong mycobacterial cell wall [5].

The Mtb cell wall is about 35 nm thick, much larger than in most other bacteria, and consists of several protective layers of varying chemical composition, including the inner lipid membrane, peptidoglycan and arabinogalactan layers, and an exceptionally tough and dense outer layer, up to 8 nm thick, that is called the outer membrane (OM) [5]. It is a bilayer membrane consisting mainly of mycolic acid molecules. Some of them are covalently linked to the arabinogalactan and peptidoglycan chains, which further anchor the membrane.

**Figure 1:**
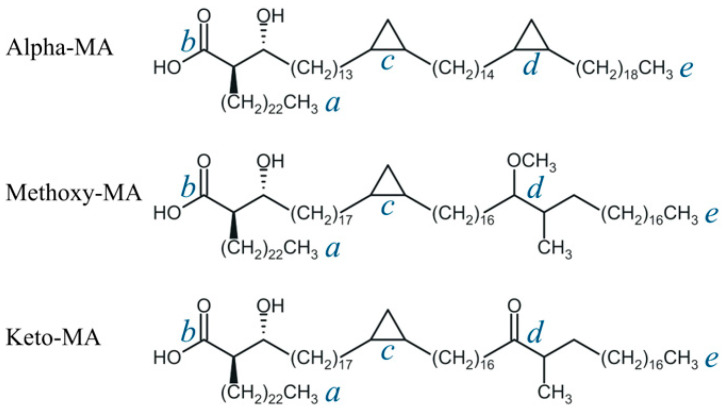
Structures of the AMA, MMA, and KMA mycolic acid molecules considered in this study. The letters *a–e* mark the key elements of the structure: *a*—end of the α-chain; *b*—carboxyl group, *c*—proximal functional group, *d*—distal functional group, *e*—end of the long hydrocarbon chain.

Mycolic acids (MAs) are the 2-alkyl-3-hydroxy long-chain fatty acids that have slightly different chain lengths and additional chemical groups among different organisms [6,7,8]. In the Mtb outer wall, the most abundant components are α-mycolic acid (AMA), ketomycolic acid (KMA), and methoxymycolic acid (MMA) (Figure 1). All of them are β-hydroxy acids with linear chains of 60–90 carbon atoms [9]. These molecules have a linear aliphatic chain of about 23 carbon atoms at the α-position with respect to the COOH group and a hydroxyl group at the β-position, corresponding to the (2R,3R) stereochemistry (fragment *a*–*b* in Figure 1). Two more functional groups are found at positions *c* and *d* inside a long hydrocarbon chain. AMA has *cis*-cyclopropane rings inserted in both the proximal (*c*) and the distal (*d*) positions with respect to the carboxyl group. It is usually the most abundant type of MA in Mtb, comprising from 50% to 70% of the total MA content [10,11,12]. MMA and KMA, collectively comprising from 30% to 50% of the total MA content, have either *cis*- or α-methyl-*trans*-cyclopropane rings inserted in the proximal (*c*) position and, respectively, methoxy and methyl or keto and methyl substituents in the distal (*d*) position [8,9].

The structure and properties of MA membranes are currently of great interest due to their protective role for mycobacteria. In particular, it was found [13] that MAs enhance Mtb resistance against chemical damage and dehydration, as well as reduce the efficiency of hydrophilic antibiotics and biocides. MAs also enable the mycobacterium to grow within the macrophages, effectively concealing it from the host immune system [11]. In our previous study [14], the structures and conformational compositions of the membranes, as well as their dynamics, were studied depending on the time and the initial membrane composition. The conformational composition has been shown to have a strong influence on membrane thickness, density, and therefore strength and permeability. In the present study, we continue the investigation of the Mtb cell wall and consider the transport of drug-like compounds through bilayer membranes of mycolic acids that provide a convenient model of the Mtb outer membrane.

The penetration of compounds through the bacterial cell wall can occur via three different mechanisms [15]:

1. Passive diffusion: This is the movement of substances directly across the lipid bilayer of a membrane from an area of high concentration to an area of low concentration that requires no energy input.
2. Facilitated diffusion: This is the movement of substances across a membrane through the special porin proteins. Porins form large, water-filled channels that allow small hydrophilic molecules to enter the cell without expending energy. This absorption pathway is suitable for a limited range of compounds because the diameter of the channel at the narrowest point determines the exclusion limit, and parameters such as the channel length and the number of open pores determine the transport rate.
3. Active transport: This is the transfer of molecules and ions against a concentration gradient, coupled to energy consumption. This process is performed by the special transporter proteins.

Experimental studies show that the outer membrane of Mtb has a very low content of porins and transporter proteins [15,16,17]. Moreover, for many (although not all) substances, penetration into mycobacteria is the better, the smaller their size and molecular weight. This allows us to assume that passive diffusion can be the main route of drug penetration through the outer membrane, at least for some drug molecules.

Direct data on the Mtb membrane permeability, both experimental and theoretical, are sparse. The experimental studies are usually conducted using the safer and more convenient model mycobacteria related to Mtb [18,19]. In the study [19], *Mycobacterium chelonei* membrane permeability was experimentally measured for eight cephalosporin-type antibiotics. Among theoretical studies, an attempt to determine the Mtb membrane permeability was undertaken by modeling the diffusion of 13 known antituberculosis drugs using the molecular dynamics (MD) method [20]. Based on the short (140 ps) simulations of a single membrane leaflet (monolayer) of pseudomycolic acid in vacuum with the MM2 force field, the diffusion coefficients and the structures of the membrane-solute complexes were investigated. 

More numerous attempts to estimate the Mtb cell wall permeability rely on the experimental data on antibiotic activity in live bacteria. An online tool for predicting the Mtb membrane permeability for small molecules was proposed based on a regression model developed by analyzing a database of antimycobacterial agents [21]. The structure-activity relationships of monocyclic nitroimidazole analogues against Mtb were investigated using docking and 3D-QSAR methods [22]. The influence of the physicochemical properties of drugs on their permeation through the Mtb membrane was also studied [23]. The proposed predictive QSAR models suggest the importance of molecular connectivity, lipophilicity, electrotopological states, and functional groups of a molecule in determining its Mtb cell wall permeability. Different machine learning models to predict the Mtb membrane permeability were compared [24]. In the study [25], a predictive in silico model of Mtb cell wall permeability applicable to diverse drugs and drug-like compounds was derived from the extensive Big Data-based dataset. However, it should be borne in mind that the machine learning and regression techniques provide only indirect estimates, and the predicted permeability can be masked by other effects, e.g., the action of the mycobacterial efflux pumps or the action of the enzymes metabolizing the drug molecules inside the living cell.

Thus, the current understanding of the factors controlling the possibility of passive transport of typical drug molecules through the outer membrane of Mtb is incomplete. In particular, there are no data on the energy barrier heights for passive diffusion. The generation of these data, their rational analysis, and the derivation of simple predictive models for the penetration barrier heights could open new avenues and accelerate the design and development of novel antituberculosis drugs. In the present study, we carry out the simulation of passive transport for a series of known antibiotics, modern antituberculosis drugs, and prospective active drug-like molecules in order to estimate the ability of potential antimycobacterial agents to penetrate through the outer mycolate membrane of Mtb. The main goal of this paper is to establish the free energy profiles, the corresponding activation barriers, and possible structural modes for the diffusing molecules. In order to estimate the free energy profile parameters, molecular dynamics simulations are employed using the all-atom force fields and the potential of mean force approximation. Various kinds of mycobacterial membranes are examined that have different chemical and conformational compositions, density, thickness, and ionization states. Based on the simulation data, we develop a series of simple regression models for the prediction of diffusion barriers from the various physicochemical properties of the penetrating molecules. Additionally, we discuss some possible mechanisms and membrane features facilitating diffusion through a membrane.

## 2. Results and Discussion

### 2.1. Selection and Appraisal of Model Drug Molecules

**Scheme 1:**
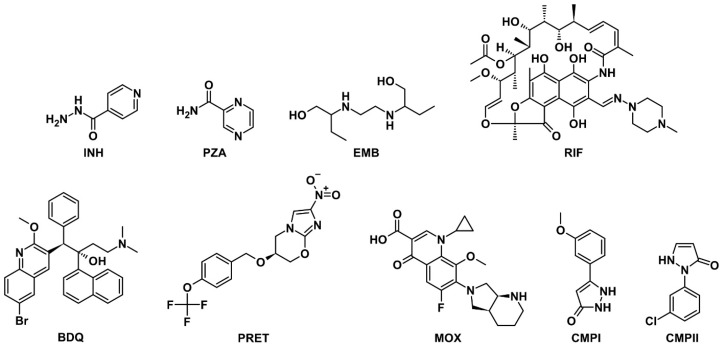
Structures of model compounds used in this study.

Several representative antitubercular drugs and drug-like compounds were selected for the diffusion study in order to cover a number of relevant scenarios. Their names, designations used in this paper, and structural formulas are presented in Table 1 and Scheme 1. In addition, Table 1 also lists the key physicochemical properties calculated for these molecules that were considered during the selection process and further investigation. Among these compounds, INH, PZA, EMB, and RIF are the classical first-line antibiotics that have been used in clinical practice for a long time, while BDQ, PRET, and MOX are relatively new and promising drugs. For the INH, PZA, EMB, RIF, and BDQ molecules, there is experimental evidence or reasonable assumptions of the passive diffusion mechanism of their transport into Mtb cells [15,26,27,28,29,30,31]. The exception is PZA, which can also cross the cell wall by several other mechanisms. The possible differences in the mode of transport between PZA and INH molecules, despite their great similarity in structure, size, and other physicochemical parameters, are not quite clear [26]. No such data are available for PRET and MOX molecules; they were considered in this work as test systems for the verification of regression models. CMPI and CMPII are compounds considered by us in our work [25]. They have been shown to have inhibitory effects on Mtb target proteins, but no antibacterial effects on live mycobacteria were found [32,33,34]. Thus, there is uncertainty about the mechanism of mycobacterial resistance to these compounds. This mechanism may lie in their inability to penetrate the cell wall or in the effect of efflux or any neutralizing systems of mycobacteria on foreign molecules. At the same time, these compounds are similar in structure to PZA and INH and are more lipophilic, which should facilitate passive diffusion across membranes. Thus, finding new data on the possible mechanism of mycobacterial resistance to these compounds represents an interesting additional topic of the present work.

### 2.2. Free Energy Profiles of Transmembrane Diffusion

The free energy profiles of transmembrane diffusion for the drug molecules in different membranes were obtained from molecular dynamics simulations using the potential of mean force (PMF) approximation (see Section 3.2, Section 3.3 and Section 3.4 for membrane designations and computational details).

**Figure 2:**
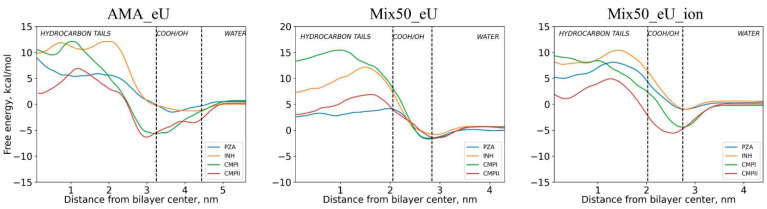
Free energy profiles for the diffusion of compounds INH, PZA, CMPI, and CMPII in neutral form through membranes of different types. Dashed lines indicate the membrane/solution interface area with the highest concentration of hydrophilic MA groups.

**Figure 3:**
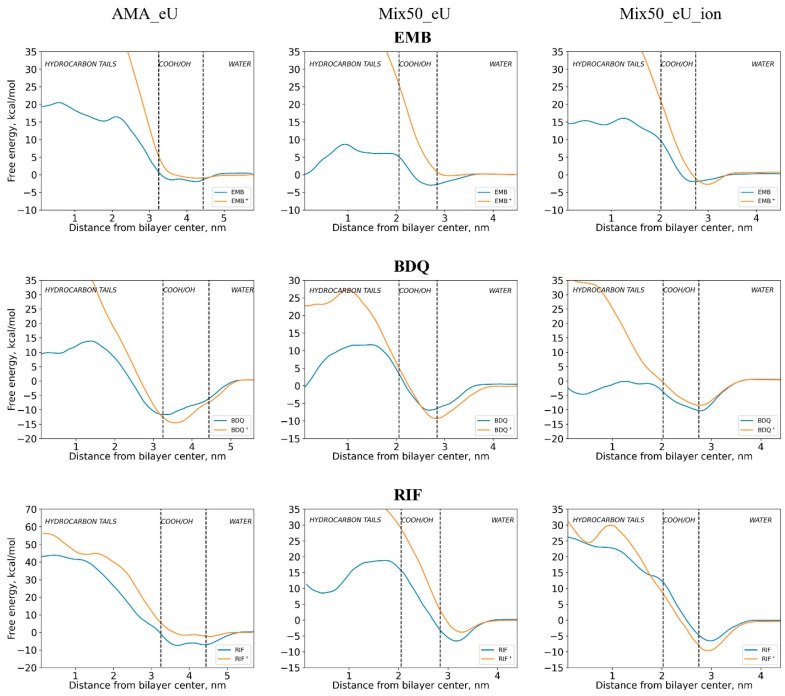
Free energy profiles for the diffusion of compounds RIF, EMB, and BDQ in neutral (blue curve) and ionized (orange curve) membranes of different types. Dashed lines indicate the membrane/solution interface area with the highest concentration of hydrophilic MA groups.

The free energy profiles of diffusion through the AMA_eU, Mix50_eU, and Mix50_eU_ion membranes for the neutral INH, PZA, CMPI, and CMPII molecules are presented in Figure 2. Figure 3 shows similar profiles for compounds EMB, RIF, and BDQ, which can exist both in neutral and ionized forms under the conditions of the considered biological systems (macrophage phagolysosome with pH = 4.5). In the free energy plots, the horizontal axis shows the distance from the center of the membrane towards its surface, and the area marked by dashed lines corresponds to the near-surface layer of the membrane that has the highest concentration of hydrophilic MA groups. The vertical axis shows the free energy of a diffusing molecule at a given point of the membrane, as determined by the PMF method. The free energy of the molecule in solution far from the membrane is taken as the zero level for these values.

It is easy to see that there is a decrease in the free energy when the diffusing molecules get into the near-surface region, which is explained by the adsorbing (and partially sorbing) ability of the surface with respect to the dissolved substance. The decrease in the energy of a diffusing molecule near the surface relative to the solution far from the surface we will conventionally call the adsorption energy, although, in contrast to the ideal adsorption, in the energy minimum region, the intrusion of the molecule into the mycolic acid layer already occurs. Interestingly, in the case of the most dense neutral membrane, AMA_eU, a markedly higher adsorption energy occurs for the CMPI and CMPII molecules compared to INH and PZA, while no such difference is observed in the case of the neutral Mix50_eU membrane, which is close in composition to the real mycobacterial membrane. This marked difference reappears when diffusing through the ionized Mix50_eU_ion membrane. A possible explanation for this is based on the different surface densities of polar groups, primarily COOH. In the AMA_eU membrane, the surface density of molecules is 3.84 nm^−2^, whereas in Mix50_eU it is 2.38 nm^−2^ [14]. For the ionized membrane, the electrostatic interactions become more significant, which favors adsorption.

The maximum of the energy profile in most cases occurs when the diffusing molecule is located in the membrane environment, moved away from the surface by 0.5–1 nm, but not yet in the center of the membrane. Near the membrane centers, in most cases, a shallow minimum is observed. For the mixed membranes, it can be explained by a decrease in density in the zone where two membrane leaflets are joined (see Supplementary Materials, Figure S1, for the membrane density profile plots). Interestingly, for the AMA_eU membrane, the density decrease in the center is not pronounced; however, a decrease in free energy occurs for all compounds except PZA (Figure 2). 

The adsorption energies and energy barriers to diffusion across the membrane are presented in Table 2. In this table, the barrier energies are presented as two values: relative to the energy in solution far from the membrane (without brackets) and relative to the adsorption minimum (in brackets). The diffusion barriers for most of the neutral compounds in different membranes are in the range of 6–25 kcal/mol (typically 15–25 kcal/mol), and only for RIF the barrier is much higher and reaches 25–50 kcal/mol. This indicates the difficulties in RIF penetration by passive transport. The diffusion barriers for the PRET and MOX molecules in the AMA_eU membrane fall into the same range as those for the compounds with experimentally confirmed transport by passive diffusion, suggesting that the same mechanism is possible for PRET and MOX. Interestingly, although the barriers for CMPI and CMPII are similar to those for PZA and INH, these two compounds show no activity in the cell-based assays. This could be explained by several possible complicating factors. First, since CMPI and CMPII, unlike PZA and INH, are not prodrugs, they could lose their activity due to metabolic reactions inside the Mtb cell. Second, the Mtb cell wall has a rather complex structure that might hinder further penetration of CMPI and CMPII into the cell in spite of the low OM diffusion barriers. Third, even after reaching the Mtb interior, CMPI and CMPII could be eliminated by the efflux pumps.

### 2.3. Influence of Molecular Charge on Diffusion Barriers

In solution, the EMB, RIF, and BDQ molecules can exist in a neutral or ionized state. From their free energy profiles (Figure 3), it is clear that the diffusion of the ionized forms is extremely difficult, reflected in the much higher energy barriers for all molecules in all studied membranes. Obviously, this is due to the fact that the ionized molecules in an aqueous solution are solvated and have to lose their solvation shells when entering the membrane. Visual analysis of the MD trajectories for ionized molecules shows that in some cases, one or two water molecules coordinated with the organic solute can be entrained and, for some time, move together with it. However, as the solute molecule penetrates deeper into the membrane (to a depth of 1–2 nm), water molecules break off and are pushed out of the membrane. The energy barriers of diffusion for the ionized forms are usually about 30–50 kcal/mol or more. In contrast, the barriers for neutral EMB and BDQ molecules are 10–30 kcal/mol, which is close to those of other neutral molecules in Figure 2. Interestingly, these values are similar even for molecules that differ greatly in molecular weight and size. A noticeable difference occurs only in the case of RIF, where the barrier is greater than 50 kcal/mol. The reasons for such disparity will be discussed below.

### 2.4. Influence of Membrane Type on Diffusion Barriers

**Figure 4:**
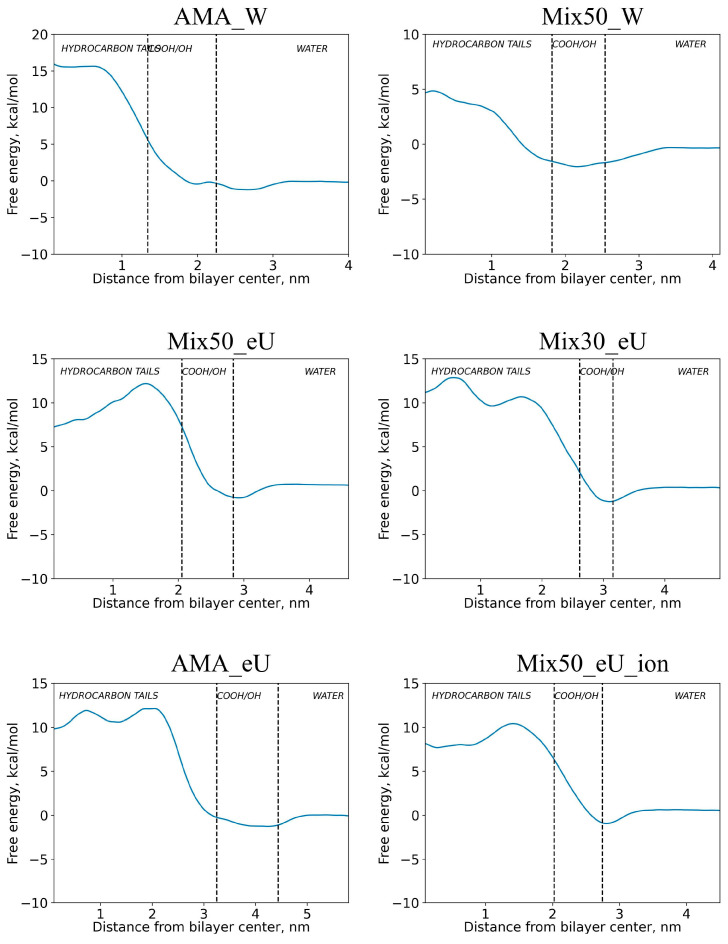
Free energy profiles of INH diffusion through different types of membranes.

Obviously, the density, thickness, and packing method of molecules inside the membrane would affect the diffusion of the molecules transported through the membrane. In Figure 4, the free energy profiles are shown for the INH molecule diffusion inside the membranes of different types. The dependence of the INH diffusion energy barrier on the membrane type is summarized in Table 3. Clearly, the diffusion barriers are related to the structure and properties of the membrane, such as its average density and spatial distribution of density inside the membrane (for the membrane density distribution plots, see Supplementary Materials, Figure S1). 

The data show that there is a marked increase in the magnitude of the energy barrier when moving from the less dense Mix50_W membrane to the more dense Mix50_eU, Mix30_eU, and AMA_eU membranes. However, the differences between the dense membranes Mix50_eU, Mix30_eU, and AMA_eU are less pronounced. Thus, although the average membrane density plays a role in counteracting passive transport, its ability to prevent the penetration of drugs into the cell has its own limits. The data also demonstrate that there is no appreciable dependence of the diffusion barrier on the membrane thickness. Thus, the membrane thickness plays the role of a time factor, increasing the diffusion time but not affecting its activation energy.

Another membrane characteristic that can affect the diffusion barriers is the presence of voids and regions of reduced density formed due to the non-ideal packing of the membrane molecules. It can be evaluated by filling the membrane volume with a mesh of small (0.15 nm diameter) spheres and retaining the “free volume” (void) spheres that have no MA or water atoms in close proximity (for the detailed procedure, see Section 3.5). A typical distribution of the free volume spheres for the Mix50_eU and AMA_eU membranes is shown in Figure S2. The total volumes of void spheres in the membrane and their ratios to the membrane volume are summarized in Table 3. It can be easily seen that in the Mix50_eU membrane, the highest density of free volume spheres is concentrated near the center of the membrane between the layers. This is consistent with the overall density profile of this membrane (Figure S1), where a decrease in density is observed in the membrane center. In contrast, in the AMA_eU membrane, the free volume is distributed much more uniformly. This corresponds to the density profile where no noticeable extremes are observed in the center of the membrane.

**Figure 5:**
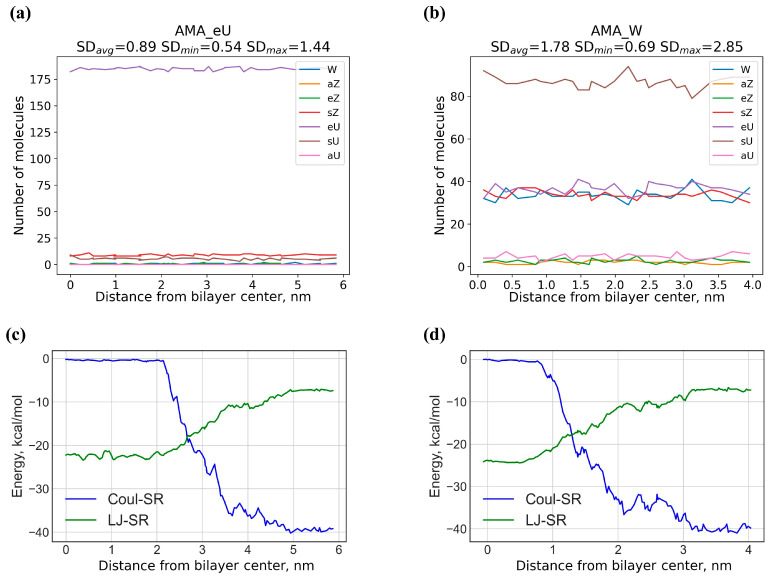
Behavior of the AMA_eU and AMA_W membranes during INH penetration towards the center of the membrane. (**a**,**b**) Changes in the conformational composition; (**c**,**d**) Changes in the short-range Coulomb and Lenard-Jones interaction energies between INH and the environment (MA, water, and ions).

Comparison of the data for AMA_W and AMA_eU in Table 3 shows that, with much lower thickness and density and, at the same time, almost the same number of voids, the diffusion barrier is much higher in AMA_W. To elucidate the reasons for this anomalous behavior of AMA_W and compare it to AMA_eU, the conformational analysis of the final systems was performed for each membrane depth of the solute. The energy contributions of the INH–environment interactions were also calculated. The results of the analysis are presented in Figure 5. The variability of the membrane conformational composition was characterized by the standard deviations (SD) of abundance levels for each MA conformation type over the range of membrane depth positions of the solute. The average, minimum, and maximum SD values for the relevant conformation types in each membrane are shown in the figure. These values indicate that the conformational changes in the AMA_W membrane during the INH permeation are about two times more pronounced than in the AMA_eU membrane. At the same time, the depth dependence of the INH–environment interaction energies for the AMA_W and AMA_eU membranes is similar. Thus, in the case of INH penetration through the AMA_W membrane, a significant contribution to the energy barrier (free energy change) is caused by the conformational transitions in the membrane, i.e., its structural instability when a drug molecule penetrates inside.

The distributions of the void spheres within the Mix50_eU and AMA_eU membranes were additionally analyzed using the DBSCAN clustering algorithm. The results of the cluster analysis are presented in Table 4. Although the amount of “noise” (spheres that could not be assigned to any cluster of the required size) in both membranes is at about the same level, their relative content is much higher in the AMA_eU membrane, indicating a larger number of uniformly distributed single and other small voids. On the other hand, Mix50_eU has a greater propensity to form clusters of significant size, as indicated by the maximum and average cluster sizes for these membranes. Thus, the general inconsistency in the changes in the Mix50_eU energy barriers for similar molecules (PZA, INH, CMPI, and CMPII) between the uncharged and charged membranes is most likely caused by the substantial roughness of the potential energy surface within Mix50_eU that is due to the non-uniform distribution of large void regions.

Attempts to find a relationship between the density of membranes of different structures and the energy of the diffusion barrier did not lead to any simple model. Although a weak general tendency to lower the barrier with increasing density is observed, the dependence has a highly nonlinear and nonmonotonic character. On the other hand, simultaneous consideration of density and free volume in the description of the diffusion barrier significantly improves the correlation. In particular, we found a linear relationship between the diffusion barrier *E*_*a*_, the average membrane density ρ, and the number of void spheres *N_void_* in the membrane, expressed by the equation.

$$E_{a}=a\rho +bN_{void}+c$$

**Figure 6:**
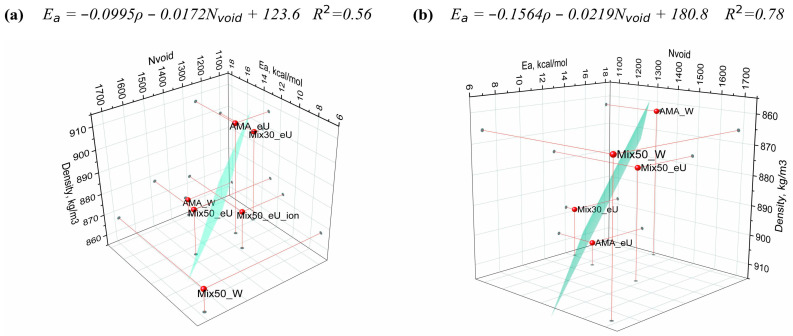
Linear models for the prediction of activation energies of INH diffusion through different membranes. (**a**) For all studied membranes; (**b**) for the neutral membranes only. Cyan rectangle in the center is the best approximating plane. Equations at the top show the best regression coefficients and the coefficients of determination.

The correlation is rather weak when the neutral and ionized membranes are considered simultaneously, but it improves significantly (*R*^2^ = 0.78) when only neutral membranes are considered. This relationship represents a plane in coordinates (*E*_*a*_, ρ, *N_void_*); it is shown in Figure 6 for the cases of all considered and all neutral membranes, along with the regression coefficients and the coefficients of determination. Although the correlations are rather weak, they allow predicting the diffusion barrier for small-molecule drugs through different types of membranes without performing an extremely expensive PMF calculation, only based on a simple MD equilibration of the membrane structure.

### 2.5. Rotational Mobility of Diffusing Molecules in Different Regions of the Membrane

One of the factors facilitating the diffusion may be the rotational mobility of the diffusing molecule, i.e., its ability to rapidly change the orientation of the molecular axes relative to the preferred alignment direction of the mycolic acid molecules. The rotational mobility of drug molecules within a membrane can characterize the ability of molecules to relax within the membrane, affecting the time to find a local energy minimum for a given molecule within the membrane and the time to stay near this minimum. To analyze this mobility, plots of the time dependence of the orientation angles of the molecules for each membrane depth were constructed (Figure S3). It can be noted that even with a slight increase in the molecule size (e.g., for CMPI and CMPII), the changes in the molecular orientation are observed much less frequently compared to the aqueous solution. Molecules of significant size (larger than PZA and INH) lose their orientational mobility compared to the aqueous solution.

### 2.6. Regression Model of Diffusion Activation Barriers Based on Solute Molecular Properties

In order to enable quick quantitative estimation of the permeability of the Mtb outer membrane, a regression model was constructed that relates the magnitude of the passive diffusion barrier of drug molecules to their physicochemical parameters. We considered the diffusion through the AMA_eU membrane, which has more uniform distributions of density (Figure S1) and free void volume (Section 2.4). Thanks to this uniformity, one can expect an essentially uniform lateral distribution of properties within the membrane (along the OX and OY axes), in contrast to Mix50_eU. Thus, the energy barriers calculated by PMF would, in this case, depend only on the transverse molecular motion along OZ, i.e., the membrane depth.

**Figure 7:**
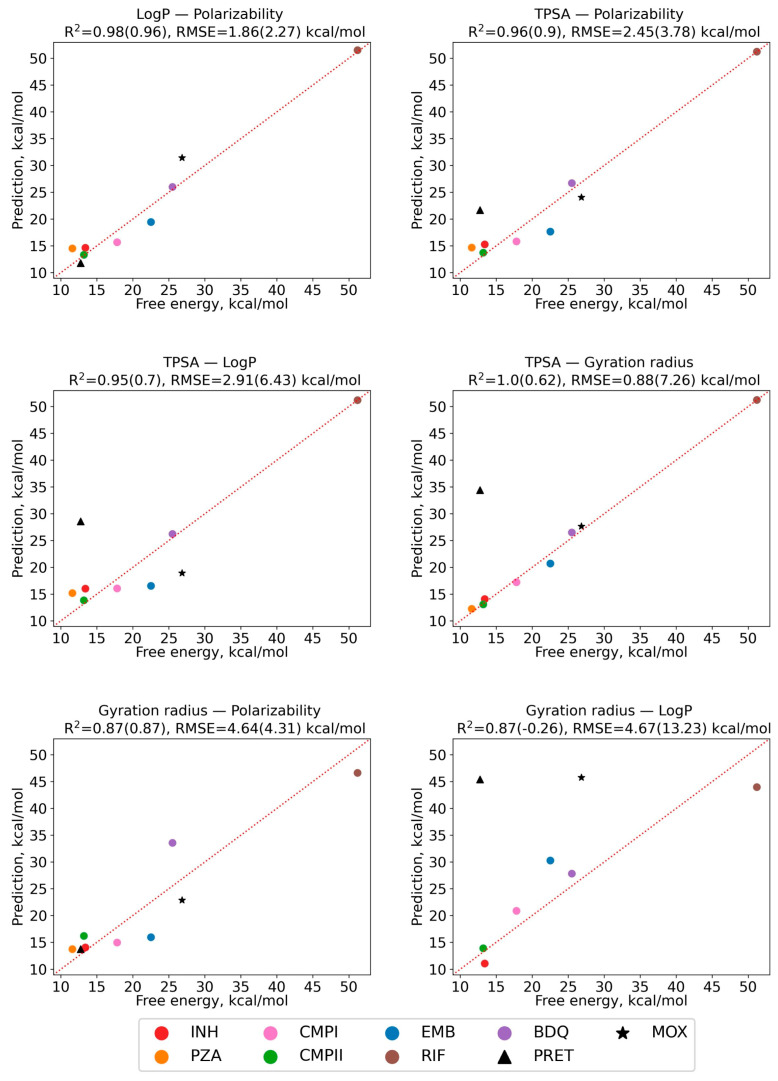
Bivariate linear regression models for the relationship between the diffusion barrier and various pairs of molecular descriptors for the solute. Horizontal axis—diffusion barrier calculated by the PMF method; vertical axis—model predictions. Black symbols denote the test set (PRET and MOX). For each plot, the coefficients of determination and RMSE values are shown for the training and full (in parentheses) data sets.

The barriers evaluated between the minimum and maximum free energy points (values without brackets in Table 2) were used as the dependent variable. The physicochemical parameters of the molecules given in Table 1 were considered as potential independent variables. All possible bivariate linear correlations were evaluated, i.e., the correlations of the dependent variable *E_a_* (predicted activation energy of passive diffusion) with all possible pairs of independent molecular descriptors. In order to verify the results, two compounds representing relatively novel classes of antitubercular drugs (PRET for nitroimidazoles and MOX for fluoroquinolones) were selected as test cases. The remaining seven compounds (PZA, INH, EMB, BDQ, RIF, CMPI, and CMPII) served as the training set to fit the model. The resulting correlations and their coefficients of determination are shown in Figure 7. The highest correlation occurs in the relationship

$$E_{a}=a\cdot cLogP+b\alpha +c$$

where *cLogP* is the predicted lipophilicity and α is molecular polarizability. The coefficient of determination (*R*^2^) of this model is 0.98 for the training set and 0.96 for the full (training + test) set. It should be noted that this model provides quite good prediction accuracy even for the test compounds, in spite of their differences. Interestingly, for the model based on the radius of gyration *R*_gyr_ and the topological polar surface area TPSA, the correlation is significantly better when considering all molecules except MOX (see Figure 7), while for the MOX molecule, the diffusion barrier determined by the PMF method is significantly lower than that predicted by the regression model. The reason for this is that the MOX molecule has TPSA and *R_gyr_* values close to those of other molecules, but its lipophilicity is drastically different. The sharp deterioration of the model when MOX is included in the test set is further evidence that a proper model should explicitly account for molecular lipophilicity. Once the best descriptor combination was established, the model parameters were refined by regression for all nine compounds (including MOX and PRET), resulting in the final regression coefficient values of *a* = −2.25, *b* = 0.63, and *c* = 3.84, with a coefficient of determination of 0.97.

The obtained linear LogP–Polarizability model is concordant with other studies revealing LogP as one of the key parameters affecting the permeation of molecules through different membranes [36,37]. In this case, the polarizability parameter also affects the interactions of the drug molecule with lipids [38]. In fact, polarizability is included in the three best models, demonstrating the importance of molecular polarizability properties in assessing the permeation of molecules through membranes, similarly to the generally accepted role of lipophilicity.

The analysis of the regression model allows one to identify and optimize the combinations of the compound properties leading to lower OM diffusion barriers. For instance, the free energy barriers tend to be low for molecules with low polarizability. The effect of large molecule size can be compensated by high LogP (e.g., for PRET and BDQ); similarly, less lipophilic molecules should have relatively small sizes (like PZA, INH, CMPI, and CMPII).

Overall, the free energy barriers for the permeation of neutral molecules through Mtb OM are generally consistent with the data for other drug molecules permeating other membranes, falling in the 5–30 kcal/mol range [39,40,41,42]. However, regardless of the lipophilicity of the molecules, OMs lack the energy profiles wherein the drug molecule would have an energy minimum inside the membrane compared to the aqueous solution [36,43,44]. There is also a clear difference in the permeation data of charged molecules through other membranes. For the charged molecules, OMs have energy barriers of 40–60 kcal/mol, while the energy barriers for lipid membranes can be relatively low [44,45]. This may be a distinguishing feature of mycolic acid membranes compared to membranes composed of lipids only. 

## 3. Materials and Methods

### 3.1. Molecular Properties of Drugs and Mycolic Acids

The physicochemical properties of the drug and MA molecules, including p*K_a_*, TPSA, polarizability, and LogP, were calculated using the Chemicalize service by ChemAxon (ChemAxon Kft., Budapest, Hungary, https://chemaxon.com/ accessed on 4 December 2023) [46]. The radius of gyration of drug molecules was calculated using RDKit 2020.03.4 software (G. Landrum, https://www.rdkit.org/ accessed on 4 December 2023) [47] on the basis of generated and thermodynamically averaged conformations. The generation of conformers (up to 20 for each molecule) and calculation of their energy (MMFF94s+ force field) were carried out using DataWarrior 5.5.0 software (Idorsia Pharmaceuticals Ltd., Allschwil, Switzerland, https://openmolecules.org/ accessed on 4 December 2023) [48]. 

### 3.2. Model Membranes

Specially constructed model bilayer mycolic acid membranes of different component compositions and thicknesses were considered in the diffusion analysis. The methods of their construction, optimization, and equilibration, as well as the conformational composition and its evolution over time, are described in detail in our previous work [14]. We denote membranes as <composition>_<conformation>, where composition is either AMA (for pure AMA membranes) or Mix30 or Mix50 (for mixed membranes with MMA + KMA comprising, respectively, 30% and 50% of the total MA content). Conformation indicates the most abundant conformation of the MA molecules at the initial state, i.e., before the MD simulations (see Section 3.5 for conformation designations). For the ionized membranes, the suffix _ion is added. Five membrane types for INH and two membrane types for all compounds from Table 1 except PRET and MOX were considered. 

The AMA_eU, AMA_W (1200 ns), 140eU–30W–30W, 100W–50W–50W, and 100eU–50W–50W outer membrane models were adopted from the previous study [14]. In the ionized membrane (Mix50_eU_ion), the carboxylate groups in the MA molecules were partially ionized according to the extreme value pH = 4.5 (observed in the phagolysosome where the mycobacterium is attacked by macrophage active compounds [49]), also taking into account the fraction of the free (not covalently linked) MAs in the OM [50]. In total, the membrane contained 40 ionized AMA molecules (20 in each leaflet). The arrangement of the ionized groups on the membrane surface was chosen so that the negative charge was evenly distributed. To this end, each membrane surface in the 100eU–50W–50W model was divided into equally spaced cells (5 along the x-axis and 4 along the y-axis). Within each cell, one randomly selected COOH group was ionized. 

The chemical and conformational composition of the studied membranes, as well as their structure and density characteristics, are presented in Table 5. The thickness and density of the membranes correspond to the state after NPT equilibration for a duration of 300 ns at 300 K in aqueous solution with Na^+^ and Cl^−^ counterions at a concentration approximately matching the physiological solution (0.15 M) [51]. The dimensions of the simulation box and the details of the MD simulations are described in the next section. Additional 40 Na^+^ ions were added to the box with the ionized membrane Mix50_eU_ion to neutralize the charges, followed by the molecular dynamics equilibration (Section 3.3).

### 3.3. Molecular Dynamics Simulations

In all MD simulations, the all-atom CHARMM36 force field [52] was used, which belongs to the modern family of force fields, continues to be actively improved, and is recommended for modeling lipid membranes [53]. The topologies for the MA and drug molecules were obtained using the CHARMM-GUI automated topology builder [54,55]. The number of atoms in the simulated systems was 140–160 thousand comprising the membrane, water (water model SPC216), and ions. Since the phagosome/lysosome contains sodium and chloride ions in a concentration approximately corresponding to a physiological solution [51], these ions were added to the system in the appropriate concentrations. The simulation was carried out in the GROMACS 2022.2 software package (GROMACS development team, https://www.gromacs.org/ accessed on 4 December 2023) [56] with the NVIDIA GeForce RTX 3080Ti GPU. 

Equilibration of the ionized 100eU–50W–50W membrane was performed in several stages: (1) energy minimization (up to Fmax ≤ 1000 kJ/mol); (2) NPT ensemble simulation with the “frozen” MA atoms (t = 100 ps, v-rescale thermostat, Parrinello–Rahman barostat, T = 310 K, pressure of 1 bar); (3) NPT ensemble simulation with “unfrozen” MA atoms (t = 100 ns, v-rescale thermostat, Parrinello–Rahman barostat, T = 310 K, pressure of 1 bar).

### 3.4. Umbrella Sampling and PMF Analysis

**Figure 8:**
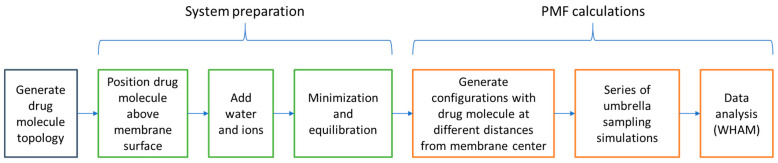
General computational workflow for the energy profile evaluation.

The umbrella sampling approach was used in combination with the potential of mean force (PMF) method to calculate the free energy profiles for the diffusing drug molecules. The general computational workflow is shown in Figure 8. The drug molecule was initially located above the center of mass (COM) of the membrane at a distance of 2 nm (inside a solution). A harmonic restraint of 2000 kJ mol^−1^ nm^−2^ and a pulling rate of 0.01 nm/ps were applied to the distance *z* between the COMs of the drug molecule and the bilayer. The reference temperature and pressure were set at 310 K and 1 bar, respectively. The temperature and pressure were controlled using the v-rescale thermostat and Parrinello–Rahman barostat. The Particle Mesh Ewald (PME) method was used to calculate the long-range Coulomb interactions. The cut-off distance for the Coulomb and van der Waals interactions was set to be 1.4 nm. Every configuration was simulated for 20 ns, and the last 10 ns of each MD run was used to calculate the free energy profile using the weighted histogram analysis (WHAM) method [57] embedded in GROMACS.

### 3.5. Analysis of MD Trajectories

The classification of the mycolic acid conformations, following the approach [12,14], was based on the distances between points *a*, *b*, *c*, *d*, and *e* located at the ends of the aliphatic chain and on the atoms of the functional groups (Figure 1). Within the WUZ classification, different arrangements on the plane for the four fragments between these points (i.e., different ways of chain bending) create seven types of conformations: W, aZ, sZ, eZ, aU, sU, and eU. Conformational analysis of MA was carried out using the k-means clusterization algorithm, according to the previous publication [14]. The structures of all MA molecules in each frame were processed using the open-source, community-developed PLUMED library version 2.7.2 (PLUMED development team, https://www.plumed.org/ accessed on 4 December 2023) [58,59] to calculate the distances between the points *a*, *b*, *c*, *d*, and *e* selected to match certain reference atoms in the MA molecules. 

To analyze the voids inside the AMA_eU and 100eU–50W–50W membranes, a script was written in Python 3 using the numpy [60], scipy [61], and pandas libraries. The entire volume of the membrane in the simulation box was filled with spheres of 0.15 nm diameter located at the nodes of the 0.15 × 0.15 × 0.15 nm^3^ mesh. Next, all spheres that had a distance less than 0.2 nm to any MA atom or a distance less than 0.3 nm to any water atom were removed. Cluster analysis of void spheres was performed using the DBSCAN [62] algorithm implemented in scikit-learn [63]. The parameter eps = 0.52 was chosen as the size of the diagonal of a cube with an edge of 0.3 nm, which corresponds to the size of two cells of 0.15 nm in this system. The minimum number of points for cluster formation was chosen to be 6 = 2 × N, where N is the number of dimensions.

To determine significant parameters of the drug molecule for permeation through the AMA_eU membrane and to construct predictive regression models, linear regression analysis was performed for each pair of variables using the LinearRegression method in scikit-learn [63]. 

## 4. Conclusions

In this study, we used the PMF approach to estimate the diffusion barriers across the outer mycolate membrane of Mtb for nine drug compounds. While most of the neutral compounds have barriers ranging from 6 to 25 kcal/mol, RIF faces higher barriers of 25–50 kcal/mol, indicating challenges for passive transport. The barriers for PRET and MOX in the AMA_eU membrane suggest potential passive diffusion.

In terms of membrane properties, this study reveals that its thickness has no significant effect on the barrier height, while the average membrane density influences it in a complex manner. A bivariate linear model considering density and free volume effectively predicts the activation barriers for neutral membranes.

Based on the PMF data, regression models were developed that offer a quick way to estimate the Mtb outer membrane permeability using easily accessible molecular descriptors. The best correlation is achieved by a bivariate linear model that links the free energy barrier to the lipophilicity and polarizability parameters, emphasizing the importance of polarizability in assessing membrane permeation. The analysis of the model can suggest combinations of compound properties leading to lower OM diffusion barriers. 

Overall, these findings demonstrate the suitability of the PMF-based approach to the evaluation of diffusion energy profiles as well as enhance our understanding of the Mtb outer membrane structure and dynamics. In the future, this could facilitate the development of novel generations of antimycobacterial drugs.

## Figures and Tables

**Table 1:** Designations and physicochemical characteristics of model compounds used in this study.

Compound	Designation	Molar Mass, g/mol	Gyration Radius, nm	TPSA, Å^2^	LogP [35] (cLogP)	Polarizability α, Å^3^
Isoniazid	INH	137.1	0.23	68.0	−0.71 (−0.69)	13.7
Pyrazinamid	PZA	123.1	0.20	68.9	−0.71 (−1.22)	11.4
Etambutol	EMB	204.3	0.34	64.5	−0.12 (−0.06)	23.4
Rifampicin	RIF	823.0	0.48	220.2	3.85 (3.50)	85.5
Bedaquilin	BDQ	555.5	0.47	45.6	6.37 (7.13)	61.9
Pretomanid	PRET	359.3	0.50	88.7	(4.14)	28.8
Moxifloxacin	MOX	401.4	0.41	82.1	(−0.49)	39.6
MOPP ^a^	CMPI	190.2	0.30	57.9	(0.33)	19.3
CPMP ^b^	CMPII	208.6	0.29	32.7	(1.61)	20.9

Note: ^a^ 5-(3-methoxyphenyl)-1H-pyrazol-3(2H)-one; ^b^ 1-(3-chlorophenyl)-3-methyl-2-pyrazolin-5-one.

**Table 2:** Characteristics of the free energy profiles of drug diffusion through the model membranes.

Diffusing Molecule	Energy of Adsorption at the Membrane Surface Layer *E*_ads_ Relative to the Bulk Solution, kcal/mol			Free Energy Barrier Relative to Minimum Point *E_a_* of Energy Profile (Relative to Bulk Solution *E*^0^*_a_*), kcal/mol		
	AMA_eU	Mix50_eU	Mix50_eU_ion	AMA_eU	Mix50_eU	Mix50_eU_ion
Neutral forms						
INH	1.3	1.5	1.5	13.4 (12.1)	13 (11.5)	11.4 (9.9)
PZA	1.7	1.5	1.4	11.6 (9.6)	5.7 (4.2)	9.1 (7.7)
CMPI	4.2	2.4	4.2	17.8 (11.4)	17.1 (14.8)	14.1 (9.9)
CMPII	3.8	2.0	5.6	13.2 (6.7)	8.4 (6.3)	10.5 (4.8)
EMB	2.4	3.0	2.2	22.5 (20.1)	11.6 (8.6)	18.0 (15.7)
RIF	7.3	6.8	6.5	51.2 (43.6)	25.4 (18.6)	33.3 (26.9)
BDQ	9.2	7.4	10.9	25.5 (13.5)	18.6 (11.2)	10.2 (−0.7)
Ionized forms						
EMB^+^	0.9	0.4	3.5	63.3 (62.4)	62.8 (62.5)	65.8 (62.3)
RIF^+^	2.3	3.7	9.3	58.6 (56.3)	48.2 (44.4)	41.4 (32.2)
BDQ^+^	13	9.1	8.9	64.3 (49.4)	36.6 (27.4)	44.8 (36.0)

**Table 3:** Dependence of the energy barrier for INH diffusion through various kinds of model membranes on the membrane properties.

	AMA_W	Mix50_W	Mix50_eU	Mix30_eU	AMA_eU	Mix50_eU_ion
Thickness *d*, nm	4.40	4.50	5.00	5.90	7.80	5.10
Average density ρ, kg/m^3^	863	866	877	898	907	873
Number of void spheres, *N_void_*	1315	1679	1477	1098	1234	1285
Volume of membrane ^a^ *V_m_*, nm^3^	407.2	427.8	420.4	411.2	404.4	427.0
Fraction of free volume, %	0.59	0.70	0.64	0.49	0.54	0.54
Diffusion barrier *E_a_ (E*^0^*_a_)*, kcal/mol	18.1 (17.0)	6.9 (5.6)	13.0 (11.5)	14.1 (12.5)	13.4 (12.1)	11.4 (9.9)
Adsorption energy *E*_ads_, kcal/mol	1.1	1.2	1.5	1.6	1.3	1.5

Note: ^a^ Calculated as $V_{m}=abd=m/\left(\rho d\right)$, where *a*, *b*—simulation box sizes in the directions parallel to the membrane surface (lateral); *m*—total mass of membrane molecules.

**Table 4:** Cluster analysis results for void spheres in AMA_eU and Mix50_eU membranes.

	AMA_eU	Mix50_eU
Total number of void spheres	1234	1477
Number of void spheres not assigned to any cluster	559	547
Ratio of the non-clustered void spheres to the total void spheres, %	45	37
Number of clusters	53	50
Maximum cluster size	58	132
Average cluster size	13	19

**Table 5:** Composition of model membranes and their properties before the PMF calculations.

Membrane	AMA_W	AMA_eU	Mix30_eU	Mix50_eU	Mix50_W	Mix50_eU_ion
Number of MA molecules in a box AMA:KMA:MMA	200:0:0	200:0:0	140:30:30	100:50:50	100:50:50	100:50:50
Initial conformations of AMA:KMA:MMA	W:–:–	eU:–:–	eU:W:W	eU:W:W	W:W:W	eU:W:W
Time of equilibration, ns	1200	300	300	300	300	100 ^a^

Note: ^a^ Started from the Mix50_eU structure equilibrated during 300 ns.

## Data Availability

Data are contained within the article or Supplementary Materials.

## Supplementary Materials

The supporting information can be downloaded at: https://www.mdpi.com/article/10.3390/ijms25021006/s1.
