# Supplementary material for: Free Energy Barriers for Passive Drug Transport through the Mycobacterium tuberculosis Outer Membrane: A Molecular Dynamics Study
Source: Int J Mol Sci. 2024 Jan 13;25(2):1006. doi: 10.3390/ijms25021006 (PMC10815926; doi:10.3390/ijms25021006)
Supplement: Supplementary file 1 [file ijms-25-01006-s001.zip › ijms-2782873-supplementary.pdf]

International Journal of *Molecular Sciences*

MDPI

Article

Free Energy Barriers for Passive Drug Transport through the *Mycobacterium tuberculosis* Outer Membrane: A Molecular Dynamics Study

Ilya S. Steshin [1], Alexander V. Vasyankin [1], Ekaterina A. Shirokova [1], Alexey V. Rozhkov [1], Grigory D. Livshits [1], Sergey V. Panteleev [1], Eugene V. Radchenko [1,2], Stanislav K. Ignatov [1,*] and Vladimir A. Palyulin [1,2,*]

[1] Department of Chemistry, Lobachevsky State University of Nizhny Novgorod, Nizhny Novgorod 603022, Russia; ilya.steshin@icloud.com (I.S.S.), alexandrvasyankin@gmail.com (A.V.V.), ekashirokova@gmail.com (E.A.S.), rozhkov@chem.unn.ru (A.V.R.), grigory.livshits@gmail.com (G.D.L.), pasv1984@gmail.com (S.V.P.) ; genie@qsar.chem.msu.ru (E.V.R.)

[2] Department of Chemistry, Lomonosov Moscow State University, Leninskie Gory 1/3, Moscow 119991, Russia

* Correspondence: ignatov@unn.ru (S.K.I.), vap@qsar.chem.msu.ru (V.A.P.)

Supplementary Materials

Figure S1 2
Figure S2 3
Figure S3 4

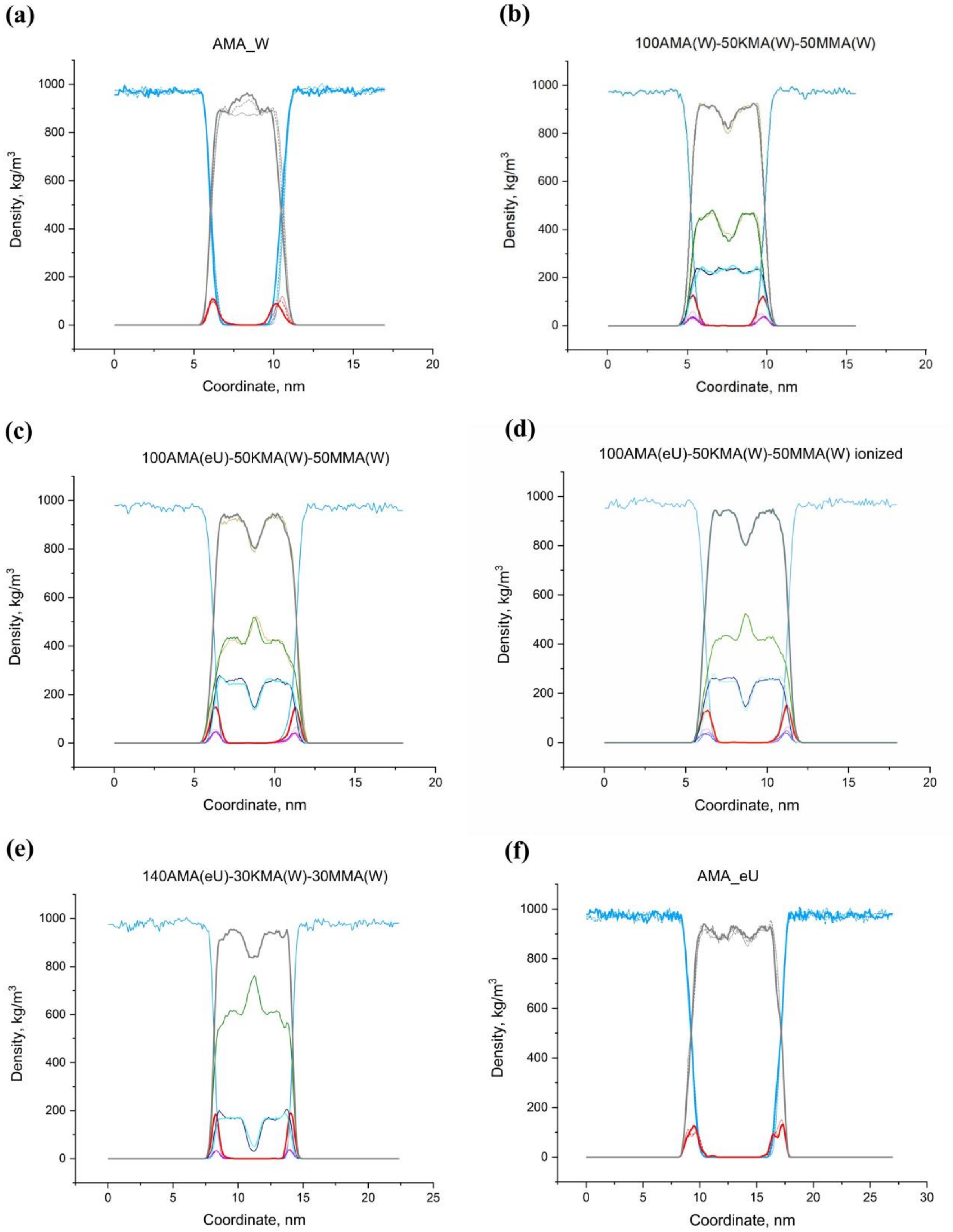

Figure S1. Density profiles for different kinds of membranes considered in the current study. *(a)* AMA_W; *(b)* Mix50_W; *(c)* Mix50_eU; *(d)* Mix50_eU_ion; *(e)* Mix30_eU; *(f)* AMA_eU. Color lines: blue – water; red – oxygens of surface groups; grey – total density of MA molecules; green – AMA molecules; magenta and cyan – KMA and MMA, respectively. Thick lines designate the final state of membranes after equilibration; thinner lines – after 100 ns of equilibration.

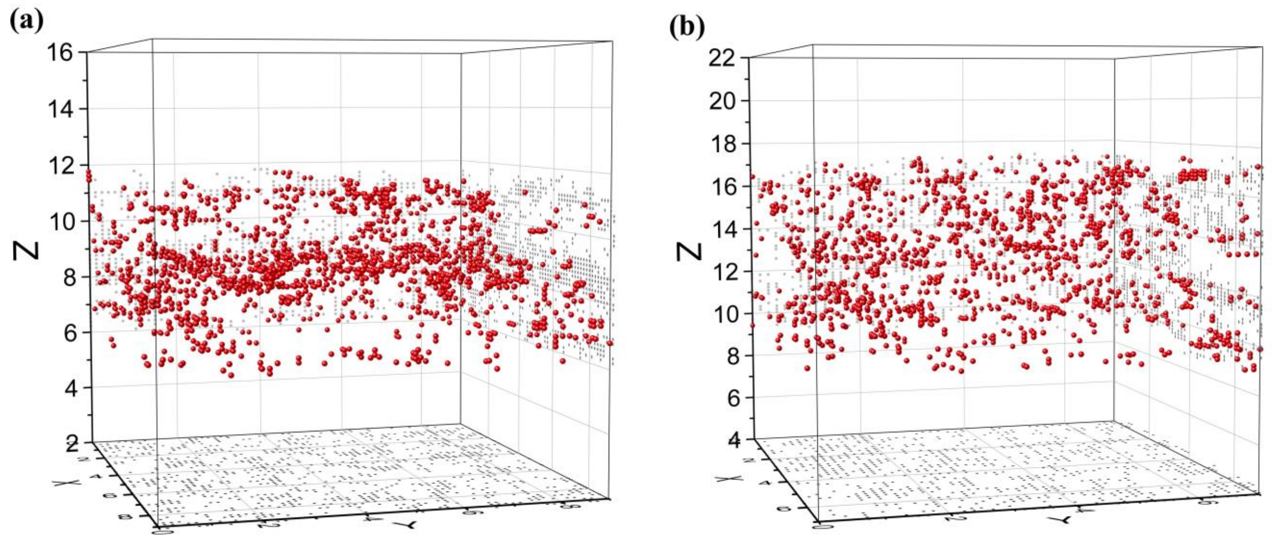

Figure S2. Free volume distribution in membranes: *(a)* Mix50_eU; *(b)* AMA_eU. Black points are the projections of free volume spheres on the coordinate planes. For clarity, the diameter of the spheres is increased compared to their real size of 0.15 nm.

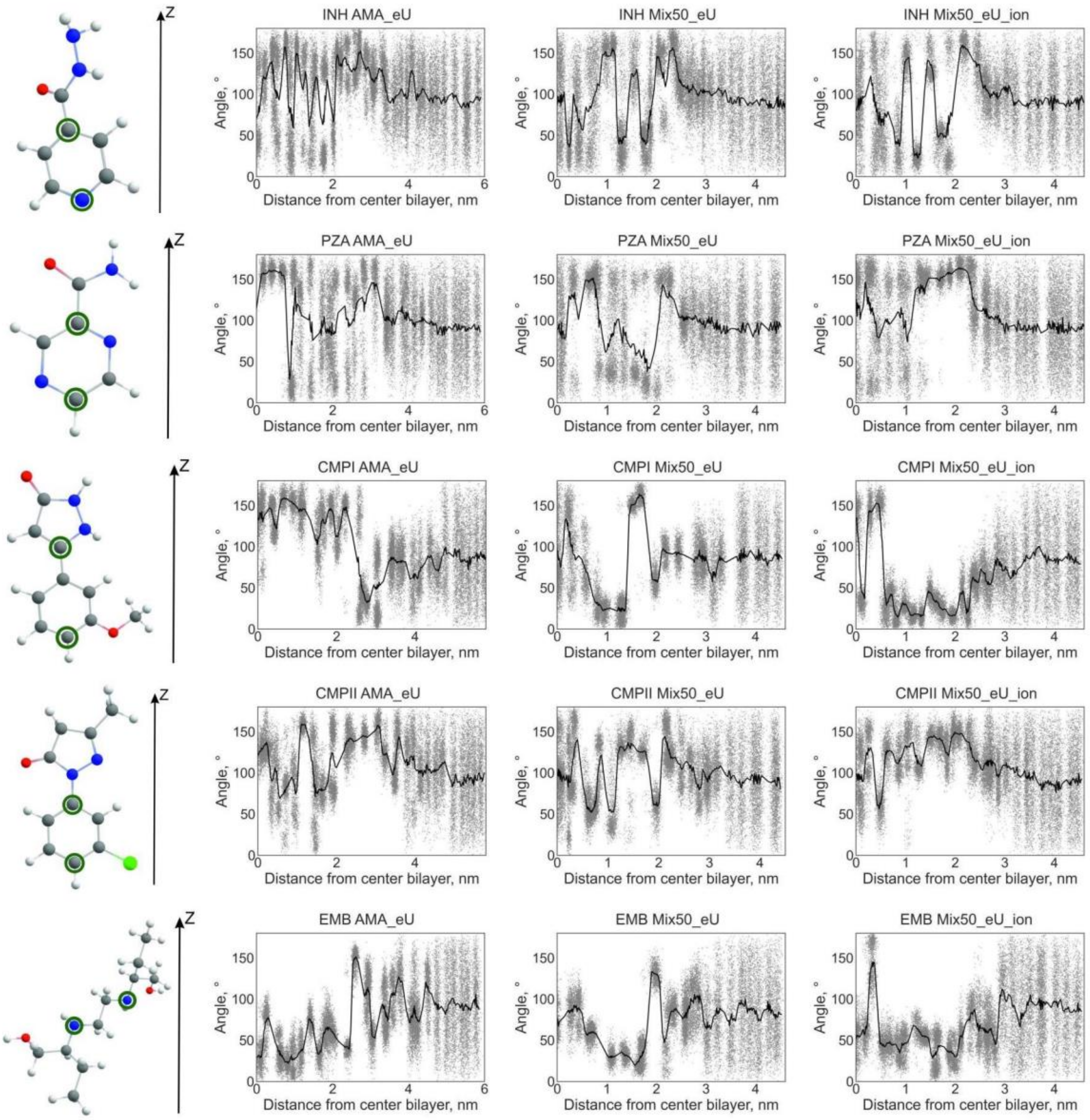

(Continued on the next page)

Figure S3. Orientation angles of diffusing molecules (left column) inside the AMA_eU, Mix50_eU, Mix50_eU_ion membranes. Horizontal axis represents the *z* coordinate of the center of mass of the molecule relative to the center of the membrane; vertical axis represents the angle between the segment connecting the reference atoms (shown as circles in the molecule formulas) and the Oz axis. Gray dots are the instantaneous angle values. The solid line is the average value of the angle at a given *z* obtained using the moving average method with a window of 150 points.

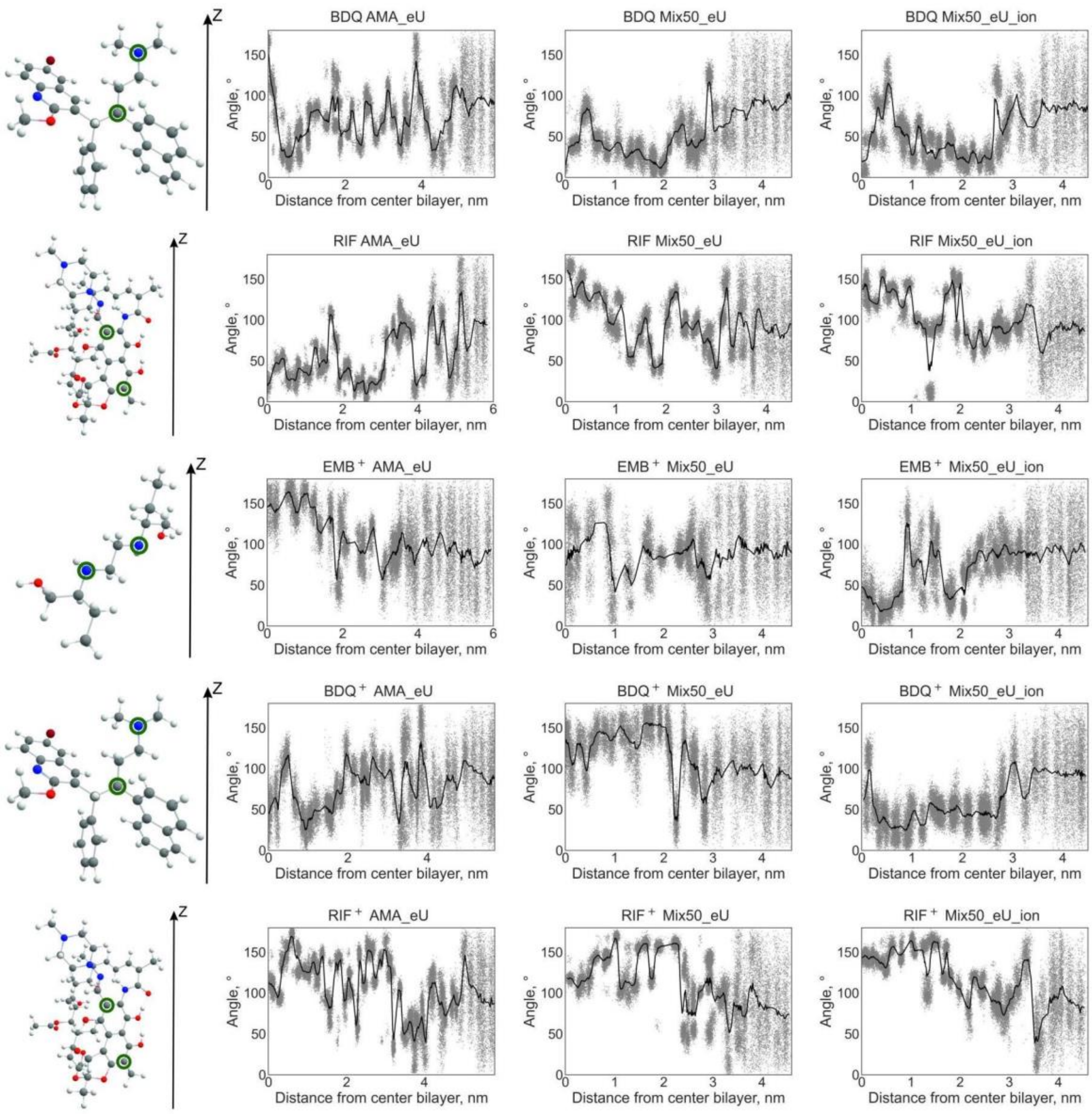

Figure S3 (continued). Orientation angles of diffusing molecules (left column) inside the AMA_eU, Mix50_eU, Mix50_eU_ion membranes. Horizontal axis represents the *z* coordinate of the center of mass of the molecule relative to the center of the membrane; vertical axis represents the angle between the segment connecting the reference atoms (shown as circles in the molecule formulas) and the Oz axis. Gray dots are the instantaneous angle values. The solid line is the average value of the angle at a given *z* obtained using the moving average method with a window of 150 points.
